# Modelling bush encroachment dynamics using Intensity Analysis and the Cellular Automata model

**DOI:** 10.1007/s10661-025-13808-x

**Published:** 2025-03-17

**Authors:** Ntuthuko Prosperous Mncwabe, John Odindi, Trylee Nyasha Matongera, Onisimo Mutanga

**Affiliations:** 1 Discipline of Geography and Environmental Science, School of Agricultural Earth and Environmental Sciences, University of KwaZulu-Natal, Scottsville, Pietermaritzburg, 3209 South Africa; 2 Centre for Transformative Agricultural and Food Systems, School of Agricultural, Earth and Environmental Sciences, University of KwaZulu-Natal, Scottsville, Pietermaritzburg, 3209 South Africa

**Keywords:** Remote sensing, Shrub expansion, Vegetation dynamics, Land cover change, Nature reserve

## Abstract

Bush encroachment is a globally recognized phenomenon linked to adverse effects, including the degradation of grasslands and loss in biodiversity, thereby challenging the conservation of keystone and flagship species, the recreational value of landscapes and local livelihoods. Therefore, a comprehensive analysis of bush encroachment is essential to gain insights into its past, present and future encroachment, as well as the severity of transitions. Using RapidEye and PlanetScope satellite imagery, this study adopted Intensity Analysis to examine past and current bush encroachment trends for the periods 2009–2014, 2014–2019 and 2019–2023, while the Cellular Automata (CA) model was used to project future encroachment trends for 2028 and 2033 within a protected area. The results indicated a continuous increase in bush encroachment within the study area. Analysis of land cover intensities shows an intensive change in the research area’s land cover in the first period (2009–2014) compared to subsequent periods. In the first two periods (i.e. 2009–2014 and 2014–2019), woody vegetation gains were more pronounced at the expense of grasslands. However, during the 2019–2023 period, woody vegetation gains were less intensive to grasslands. Moreover, throughout the study period, most grassland gains occurred in bare areas, whilst the primary cause of grassland losses was bush encroachment. The projection of future encroachment trends indicates a continued increase in woody vegetation over the next decade. The results also indicate that bush encroachment is projected to expand by 5.50 and 6.67% in 2028 and 2033, respectively. These findings highlight the urgent need to assess and enhance management schemes within the study area. Gaining critical insights into bush encroachment progression trends and transition intensities can help prioritise landscape management efforts and support decision-making for the restoration of grasslands.

## Introduction

Bush encroachment is a global phenomenon that occurs when grass-dominated landscapes are encroached by bushes, resulting in the suppression of palatable grasses (Belayneh & Tessema, 2017; Yassin, 2019). In Southern Africa, for instance, bush encroachment is prevalent and affects approximately 0.131 to 1.275% of the landscape per year (O’Connor et al., 2014). Stafford et al. (2017) reported that bush-encroached areas cover about 10–20 million ha of South African grasslands and savannas, whilst more recently, Mndela (2020) highlighted that bush encroachment threatens approximately 7.3 million ha of the entire South African land area.

Bush encroachment is particularly severe in protected areas such as nature reserves (Hudak & Wessman, 2001). These landscapes hold significant conservation and recreational value due to their rich diversity of animal and plant species (Xu et al., 2020). However, in most cases, due to injudicious management practices, such as infrequent fires and inconsistency of mechanical control methods, the majority of nature reserves are commonly susceptible to bush encroachment (Shekede et al., 2015). In these landscapes, bush encroachment threatens various ecosystem functions, biodiversity of flora and fauna, recreational opportunities and local livelihoods (Mogashoa et al., 2021; Nakanyala & Hipondoka, 2020; Wiegand et al., 2006). The intrusion of woody vegetation in nature reserves also modifies the landscape, increasing its susceptibility to soil erosion. Additionally, it affects nutrient cycling and landscape productivity (Hudak & Wessman, 2001; Stewart et al., 2022). Moreover, bush encroachment limits the diversity of habitat structure and impacts the quantity and quality of land suitable for grazing (De Klerk, 2004). Consequently, bush encroachment increases the reserve’s vulnerability to grazing pressures, disrupts wild herbivore densities, reduces the reserve’s carrying capacity and increases management costs (Ayelew & Mulualem, 2018; O’Connor et al., 2014; Stafford et al., 2017). Given these threats, it is important to understand the progression, trends and spatio-temporal patterns of this phenomenon.

Monitoring and understanding bush encroachment is vital for the protection and management of nature reserves (Ben-Shahar, 1992). In this regard, the availability of innovative technologies such as remote sensing allows for precise analysis of bush encroachment at various spatial extents (Maphanga et al., 2022). Remote sensing technologies facilitate the process of acquiring explicit information on spatio-temporal changes of bush encroachment, contributing to decision-making for the management of nature reserves (Graw et al., 2016; Ludwig et al., 2016). Specifically, the availability of high spatial resolution sensors opens new research opportunities for improved local-scale landscape delineation and assessment of land cover transitions, necessary for improving the evaluation of landscape vulnerability to bush encroachment. For instance, datasets such as RapidEye and PlanetScope have high spatial resolutions of 5 and 3 m, respectively, and provide detailed spatial and spectral information valuable for the assessment and mapping of vegetation health and environmental changes (Gašparović et al., 2018; Roessler et al., 2013). The inclusion of RapidEye’s additional red-edge band, for instance, enhances sensitivity to chlorophyll content changes, facilitating improved mapping and discrimination of vegetation types (Kim & Yeom, 2015; Marx & Tetteh, 2017; Zhang et al., 2021).

The value of high spatial resolution image datasets has been shown in various vegetation mapping and modelling applications (Adam et al., 2014; Balha et al., 2021; Khare et al., 2018; Neyns & Canters, 2022). However, for a detailed understanding of bush encroachment, effort beyond transition mapping is critically important (Akinyemi et al., 2017; Xie et al., 2020). Hence, methods such as Intensity Analysis can be utilised to comprehensively understand the change processes associated with bush encroachment and to quantify the dynamics of landscape transitions (Osman et al., 2023). Intensity Analysis is a mathematical framework that has been extensively utilised for land use and land cover analyses at three levels, namely, interval, categorical and transition (Pontius et al., 2013). The method has been adopted in China (Zhou et al., 2014), Ghana (Ekumah et al., 2020), Iran (Kourosh Niya et al., 2019) and Kenya (Osman et al., 2023) to offer insights into the dynamics, drivers and impacts of land cover changes. Zhou et al. (2014), for instance, employed Intensity Analysis to evaluate land cover changes in a coastal watershed of southeast China at interval, categorical and transition levels. The study showed that land use transformation has been accelerating over the years and that agricultural activities were intensive throughout the study period.

Intensity Analysis is particularly valuable in assessing annual change area and change intensity of land cover transitions over different periods by comparing the observed with the uniform changes (Aldwaik & Pontius, 2012; Xie et al., 2020). Specifically, uniform changes are estimated changes that would exist if the transition were distributed uniformly across the landscape, and observed changes are the actual changes that are happening in the landscape. Intensity Analysis is particularly useful for comprehending gains and losses to a specific land cover class (Feng et al., 2020). For instance, it can be used to evaluate why the area transition from land cover class A to class B is greater than the transition from other land cover classes (Quan et al., 2020). Moreover, Intensity Analysis has the potential to establish the extent of bush encroachment and provide information on active and dormant categories, revealing the stationarity of processes and patterns of change (Huang et al., 2012). The active land cover category is the category that has its gains or losses exceeding the estimated landscape changes, whilst the dormant category is a category with gains or losses that are lower than the estimated landscape changes. Generally, the Intensity Analysis presents an opportunity for an in-depth analysis of bush encroachment and can substantially enhance our understanding of the phenomenon at various spatial extents. Its insights can also support land management strategies, conservation efforts and sustainable development in nature reserves and other protected areas (John et al., 2013).

Reliable assessment of bush encroachment and optimal management of protected areas could also be facilitated by the prediction of future encroachments and landscape changes (Liao et al., 2018; Taylor et al., 2018). Based on accurately classified land cover change maps, studies have simulated future bush encroachment patterns to understand the dynamics and severity of the phenomenon in grasslands and other bush-encroached landscapes (Cao et al., 2019; Caracciolo et al., 2014). The prediction of future encroachments is particularly useful for analyses of ecosystem vulnerability to bush encroachment and various related phenomena such as overgrazing, land degradation and the effects of climate change (Liao et al., 2018). Furthermore, land cover simulation models are essential for providing valuable information for long-term management planning (Gaur & Singh, 2023; Munthali et al., 2020). They contribute to understanding the spatiotemporal patterns of bush encroachment, helping managers develop adaptive management strategies that account for future changes in vegetation dynamics and ecosystem structure (Osman et al., 2023). Moreover, predictive models can serve as early warning systems, allowing managers to anticipate and mitigate the severity of bush encroachment (Behera et al., 2012; Melesse et al., 2007). In protected areas, prediction of future encroachments can also help optimise resource allocation by identifying priority areas for intervention through targeted efforts on areas most susceptible to encroachment (Gao et al., 2020; Wang et al., 2018). In protected areas, land cover simulation models can also be adopted to inform policy development and land use planning.

Amongst the often-utilised simulation models for land cover changes is the Cellular Automata (CA) model (Qiang & Lam, 2015; Saputra & Lee, 2019; Xing et al., 2020). The CA, a spatially dynamic model, utilises remotely sensed data and environmental variables to facilitate land cover simulation and provide detailed insights on land cover transitions (Behera et al., 2012; Yagoub & Al Bizreh, 2014). The model uses artificial intelligence algorithms such as Artificial Neural Networks (ANN) and employs a multilayer perception (MLP)-ANN learning process to replicate land cover processes and provide reliable future predictions of landscape changes (Tong & Feng, 2020). Moreover, the CA model is robust, simple to calibrate and well-adapted for spatial and temporal modelling of land cover changes in complex landscapes (Sajan et al., 2022). The CA model is popular for its reliability in modelling land cover changes (Ghalehteimouri et al., 2022; Khan et al., 2022; Koko et al., 2020). A study by Mahamud et al. (2019), for instance, employed the CA model to examine future land use and land cover outlooks in Kelantan, Malaysia. By comparing the actual 2008 Land Use Land Cover (LULC) map and the projected 2008 LULC map, the model attained an overall accuracy of 78.57%. Saputra and Lee (2019) also used the model to simulate future land cover changes for the years 2050 and 2070 in North Sumatra, Indonesia, with 87.28% accuracy and a kappa value of 83% from the comparison of reference and simulated 2010 LULC maps. The CA model was also employed to determine future land cover prediction in Dhaka, Bangladesh, with 96.62% accuracy and a kappa value of 95% attained from the comparison of simulated and reference maps of 2018 (Kafy et al., 2021).

The application of land cover simulation models, in tandem with the Intensity Analysis can be valuable to understanding the dynamics and trend processes of bush encroachment in protected areas. Consequently, studies are necessary for effective management strategies to rehabilitate grasses and prevent further encroachment. Therefore, this study sought to compute, analyse and predict spatiotemporal changes in bush encroachment from 2009 to 2023 in the Bisley Nature Reserve using the Intensity Analysis and CA model.

## Materials and methods

### Description of the study area

**Fig. 1:**
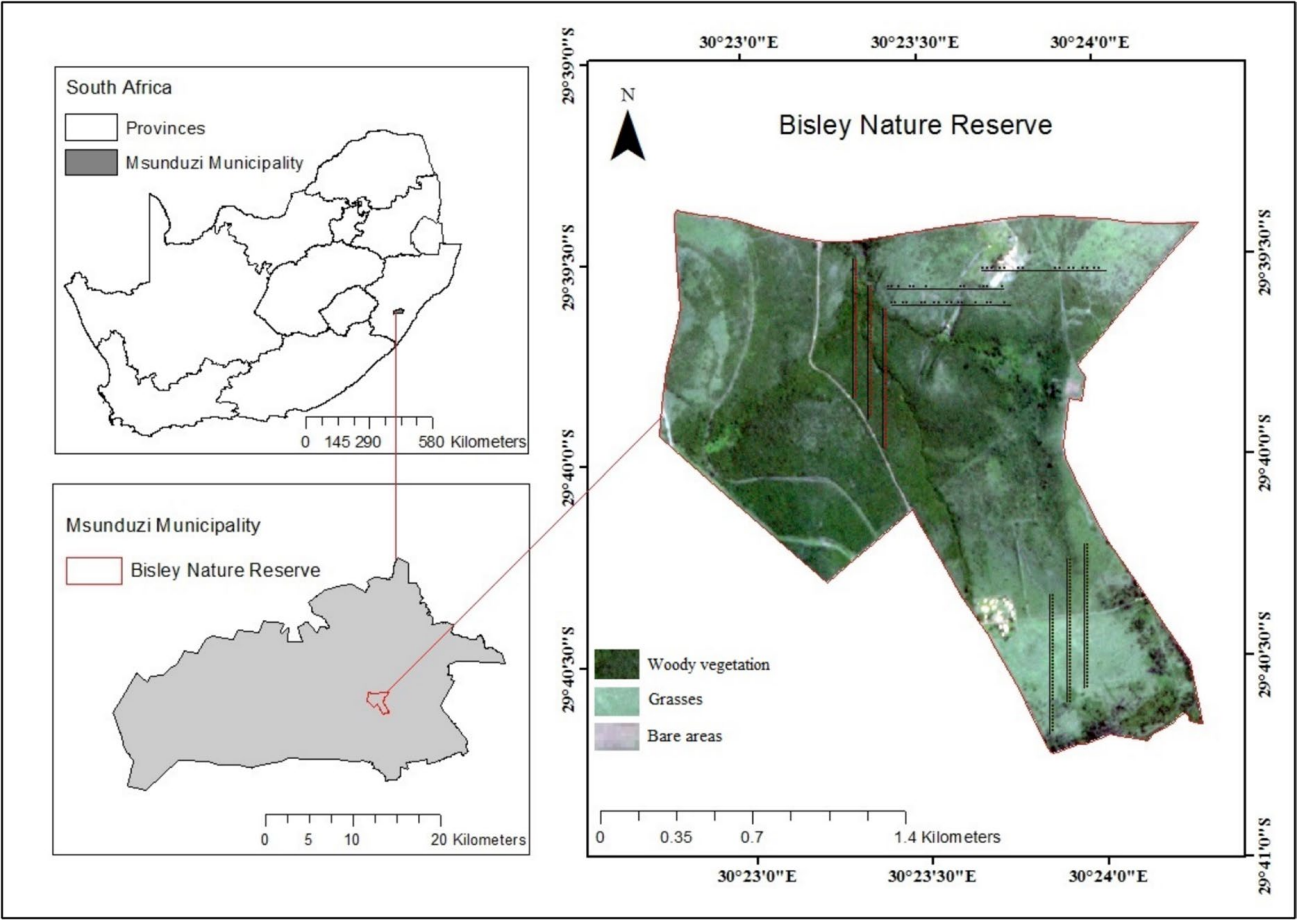
Geographic position of the experimental site in KwaZulu-Natal province, South Africa. Black lines illustrate the layout sampling transects on the landscape, using an example of three transects per land cover class. Red, green and brown points indicate locations for woody vegetation, grasses and bare areas, respectively

The study was conducted in Bisley Nature Reserve, located at Lat 29.6566, Long 30.3956, 7 km from Pietermaritzburg Central Business District (CBD) in the KwaZulu-Natal Province of South Africa (Fig. 1). The study site is situated between 720 and 840 m above sea level and occupies approximately 350 ha. Bisley has a humid subtropical climate, receives warm and humid summers, cool and dry winters and a mean rainfall of 694 mm per year (Kraai et al., 2023). The area experiences average annual temperatures of around 26 °C in summer and approximately 11 °C in the winter (Kraai et al., 2023). Shale and dolerite dominate the geological landscape, and the area has diverse soil types namely, clay loam, silt loam, sand and sandy loam. The nature reserve was established to safeguard and preserve biodiversity and to promote recreational opportunities to residents and the city of Pietermaritzburg. Bisley Nature Reserve has a range of birds and animals such as giraffes, impala, zebra, wildebeest and monkeys. The reserve is a bush-encroached grassland that is dominated by *Acacia* spp., thornvelds and shrubs. The most common grasses in the area are *Panicum maximum, Eragrostis curvula* and *Paspalum dilatatum*. In the reserve, various management strategies such as controlled burns and bush clearing have been used to manage the grasslands. However, the application of such control measures has been inconsistent.

### Data description

The study utilised two distinct datasets; environmental variables and land cover change maps. The latter encompassed the land cover classification for the years 2009, 2014, 2019 and 2023. Land cover change maps were created using RapidEye and PlanetScope images of the summer season (December, January and February) that were acquired from Planet Education and Research (https://www.planet.com/). The platform provides datasets that are already atmospherically corrected to surface reflectance. The RapidEye images were captured on 25 January 2009, 18 December 2014 and 04 February 2019, and PlanetScope was captured on 03 December 2023.

**Fig. 2:**
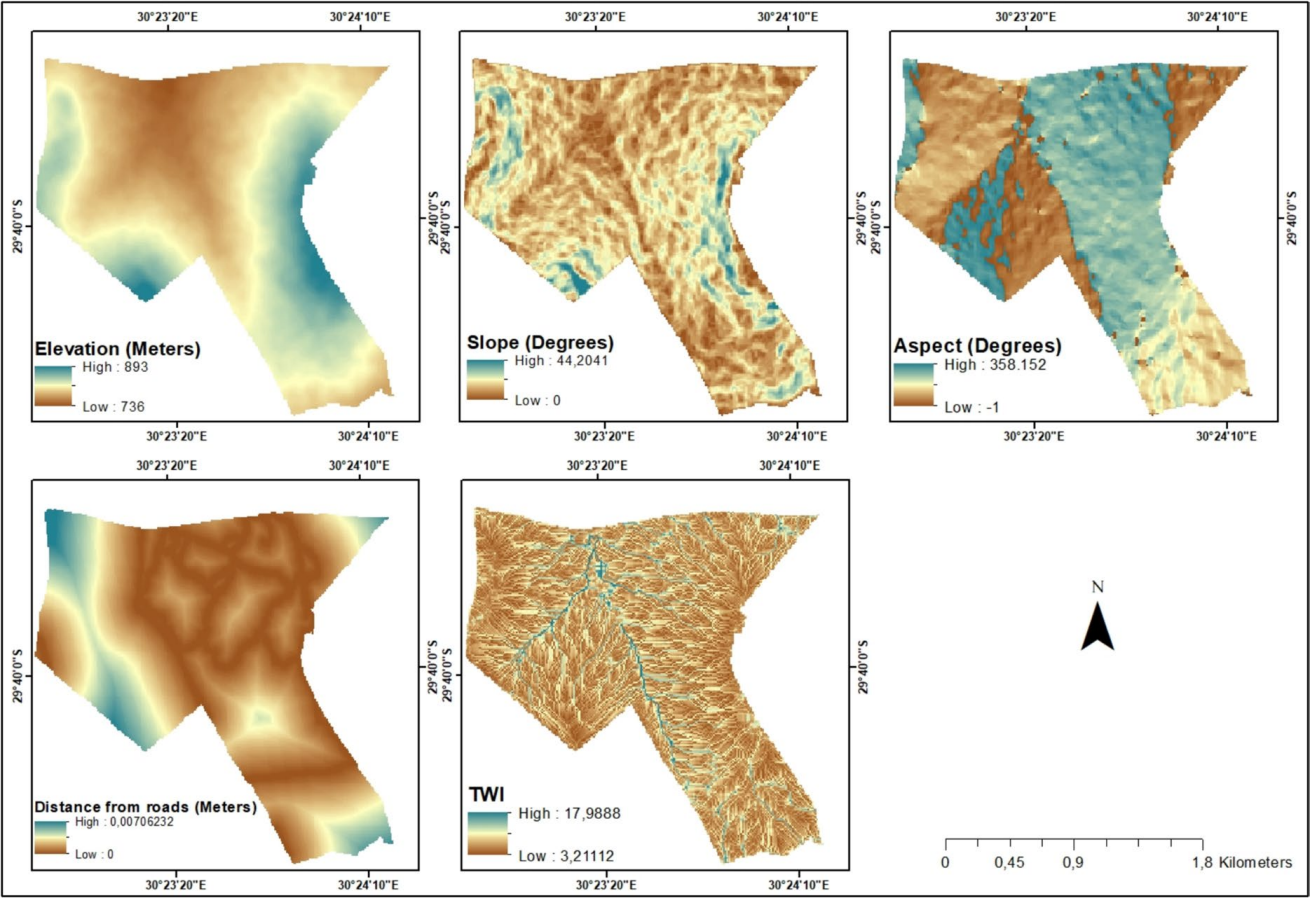
Environmental variables used for future bush encroachment prediction. TWI, Topographic Wetness Index

The environmental variable datasets included the Digital Elevation Model (DEM), slope, aspect, distance from the road and Topographic Wetness Index (TWI) (Fig. 2). Environmental variables are spatial references that are critically important in identifying the driving factors for the spatial landscape features, and they significantly influence the encroachment of woody vegetation (Liu et al., 2021). The DEM is amongst the popularly utilised datasets for analysis of land cover change and vegetation prediction, particularly due to its value in deriving other key datasets such as slope, aspect and Topographic Wetness Index (Hansen & Loveland, 2012; Singh et al., 2018). DEM offers viable information on the terrain and is critically important for the analysis of spatiotemporal changes in relation to elevation (Mishra et al., 2020). According to Wu and Archer (2005), for instance, DEM and slope affect water drainage patterns, soil erosion and nutrient distribution, which are critical for woody vegetation growth. The slope and aspect are other fundamentally important variables in LULC predictive models (Birhanu et al., 2019; Iqbal & Khan, 2014). These variables create spatial variation and influence growth patterns and distribution of vegetation (Hao et al., 2021). Aspect is also known to determine solar radiation exposure and influence vegetation distribution and bush growth (Stephen et al., 2023). The distance from the road is also a key parameter in the analysis of vegetation dynamics and acts as a proxy for human disturbances, which can impact encroachment patterns (Eldridge et al., 2013). For instance, disturbance from roads significantly impacts soil characteristics and vegetation composition and usually reduces plant diversity (Deljouei et al., 2018). Additionally, the TWI is also frequently utilised in vegetation analysis, particularly due to its strength in determining soil moisture and its influence on vegetation patterns (Hojati & Mokarram, 2016; Slezák et al., 2022). According to Tan et al. (2024), TWI is a key factor for vegetation establishment and encroachment. The 10-m spatial resolution DEM was acquired from the Alaska Satellite Facility data search vertex (https://search.asf.alaska.edu/#/) and used to derive slope and aspect. The shapefile for Bisley roads was acquired from the OpenStreetMap platform (https://www.openstreetmap.org) and used to derive data for distance from the road using the Euclidean distance tool in ArcMap 10.4 software.

### Field data collection

Field data were collected in June 2023 using a handheld sub-metre accuracy Trimble Global Positioning System (GPS). The study adopted a purposive sampling method to collect samples for woody vegetation (trees and shrubs), grasses (open areas covered with grass species) and bare areas (open areas consisting of exposed soil, with no vegetation cover). Purposive sampling provides precise acquisition of the best-suited elements of the required data. For this study, thirty transects were purposively laid based on the presence of natural vegetation. Through visualisation, the transects were distributed according to encroachment categories (i.e. heavy, medium and no encroachment) to capture variability across the study site. For each transect, a 70-m distance was measured, and a total of five sample plots were established along each transect. An area of 25 m^2^ (5 by 5 m plot) was measured for each sample plot and, a 10-m distance was established between the plots to avoid autocorrelation. A single GPS location per plot for a single land cover class was collected to indicate the position of the class in the landscape, and, in total, 150 GPS locations were collected for each land cover class (see Appendix 3).

In change detection, using the same training samples across different time periods can potentially reduce classification accuracy. Consequently, Google Earth Engine (GEE) was utilised to generate land cover samples for previous years, 2009, 2014 and 2019, using a single RapidEye image of the summer season for each of the years. Random sampling and visual interpretation were adopted for the collection of three land cover classes, namely woody vegetation, grasses and bare areas using point format. The format was preferred over polygons as it is less susceptible to the influence of spatial autocorrelation. For each period, a total of 450 sample points were collected, i.e. 150 points for each land cover class. The pre-collected 2023 land cover samples from the field campaign were imported to GEE. Image classification involved splitting the data into training and testing sets, with 70% dedicated to training the classification model and the remaining 30% reserved for model testing.

### Satellite image acquisition and pre-processing

RapidEye and PlanetScope satellite data used in this study were obtained at five-year intervals spanning from 2009 to 2023. Specifically, the study used three RapidEye images tailored for the years 2009, 2014 and 2019 and one PlanetScope image for the year 2023. All satellite image data were acquired from the Planet Education and Research online platform (https://www.planet.com/) and imported into the GEE platform for analysis. These datasets were acquired pre-processed and atmospherically corrected. RapidEye collects data across multiple frequency ranges of the electromagnetic spectrum and provides imagery with five spectral bands: red (0.63–0.68 μm), green (0.52–0.59 μm), blue (0.44–0.51 μm), near-infrared (0.76–0.85 μm) and red-edge band (0.69–0.73 μm). It features a temporal resolution of 1 day and a spatial resolution of 5 m. Additionally, its red-edge and NIR bands are particularly valuable for vegetation mapping. PlanetScope. On the other hand, offers a high temporal resolution (daily) and a spatial resolution of 3 m. It provides images with an orthorectified pixel size of 3.125 m in the red (59–670 nm), green (500–590 nm), blue (455–515 nm) and near-infrared (780–860 nm) wavelengths. Additionally, it captures images with a red-edge band, which is crucial for remote sensing of vegetation. Since PlanetScope and RapidEye sensors operate at distinct spatial resolutions, bilinear interpolation was applied to resample PlanetScope imagery and adjust its spatial resolution to match the 5-m resolution of RapidEye.

### Image classification

Three RapidEye images from 2009, 2014 and 2019, along with one PlanetScope image from 2023, were used to create land cover maps. Three land cover classes were selected: woody vegetation, grasses and bare areas. The maps were classified within the GEE platform using the Random Forest (RF) algorithm. RF algorithm is one of the most widely used machine learning algorithms for land cover classification (Adam et al., 2014; Symeonakis & Higginbottom, 2014). It is effective in handling large and noisy datasets, reducing overfitting and providing high classification accuracy compared to many other machine learning algorithms (Phan et al., 2020; Qabaqaba et al., 2023; Zhang et al., 2017).

### Change detection

Four bush encroachment classification maps were produced showing the progression of woody vegetation during the different periods. The first three classification maps represent the years 2009, 2014 and 2019, depicting the evolution of bush encroachment in a five-year interval, and the fourth map shows bush encroachment in a four-year interval. The classification maps and their corresponding transition matrices (a table showing land cover class categories and transitions in a particular period) were created by developing a code in the GEE platform. Comparisons were made between the classification maps of different time intervals to discriminate the change in the total area of woody vegetation and grasses at Bisley Nature Reserve. For instance, to compute the first transition map showing changes in the total area of woody vegetation and grasses, the 2009 and 2014 classification maps were used. The remaining three transition maps were created using the 2014 and 2019, 2019 and 2023 and 2009 and 2023 classification maps. Transition maps are maps showing alterations that occurred during the periods. Within the GEE, a code was generated using the area function and utilised for the calculation of transitions and area change between woody vegetation and grasses.

### Accuracy assessment

The assessment of the reliability of classification results was conducted using the confusion matrix. The method employs popular metrics—overall accuracy, producer’s accuracy and user’s accuracy—to assess the classified maps and provide insights on the model performance. A confusion matrix is paramount for identifying the type of errors during the classification process, such as omission and commission errors. Additionally, the kappa index was used to determine the proportion of correctly classified pixels and to evaluate both observed and expected accuracy. However, concerns have been raised about the use of kappa, as it may be misleading in practical applications and computes agreement based on chance (Feizizadeh et al., 2022; Pontius & Millones, 2011). To address this limitation, the study also used the Quantity and Allocation Disagreement (QADI) index to assess the level of agreement between the reference and classification maps (Pontius & Santacruz, 2014). The QADI index evaluates accuracy using two types of errors, namely quantity disagreement and allocation disagreement. Quantity agreement is the disparity in each class’s pixel count between the reference and classification map, whereas allocation disagreement is the count of pixels that were incorrectly identified. The QADI index values range between 0 and 1, where the value close to or equal to zero represents a low disagreement between training data and classification results, and the value of one or close to one represents a high disagreement (Pontius & Millones, 2011). The confusion matrix was utilised to compute the QADI index in ArcMap 10.8 using the Feizizadeh et al. (2022) toolbox.

### Intensity Analysis

Intensity Analysis was utilised to analyse transition maps and determine the variations of land cover change patterns across the periods. The method provides a more detailed analysis of landscape changes and links change patterns across classes with their associated processes. Intensity Analysis uses transition matrices to quantify the annual change intensity in each period at three levels, namely time interval, category and transition (Pontius et al., 2013). The assessment of time interval level calculates the size and rate of change over a certain period. Time interval level is important for determining which period has a fast and slow annual rate of overall change. For every time interval, the category level computes variations in the size and intensity of change (total losses and total gains) amongst land cover classes. Category level assesses which land cover classes are active and which are dormant in a particular period. The transition level, on the other hand, is based on the time interval and category level. The transition level deals with the amount and direction of transitions across land cover classes at each period. Moreover, the transition level evaluates if transitions are intensive or not and if they are targeted or avoided by specific land cover classes.

The Intensity Analysis across various periods of the study was conducted using a Microsoft Excel open-source programme acquired from the Intensity Analysis website (https://sites.google.com/site/intensityanalysis/). The programme computes intensities using Eqs. (1)–(6) below (Aldwaik & Pontius, 2012) (Table 1).

**Table 1:** Mathematical symbols used for calculation of intensities as reported by Aldwaik and Pontius (2012)

Symbol	Description
*Yₜ*	Year at time point *t*
*t*	Index for the initial time point of an interval [*Y*_*t*_, *Y*_*t* +1_], where t ranges from 1 to *T* − 1
*J*	Number of categories
*i*	Index for a category at the initial time point of an interval
*j*	Index for a category at the latter time point of an interval
*n*	Index of the gaining category for the selected transition
*Sₜ*	Annual change during the interval [*Yₜ*, *Y*_*t* +1_]
*U*	Uniform annual change during extent [*Yₜ*, *Yₜ*]
*G*_*tj*_	The intensity of annual gain of category *j* during the interval [*Yₜ*, *Y*_*t* +1_] relative to the size of category *j* at time *t* + 1
*L*_*ti*_	The intensity of the annual loss of category *i* during the interval [*Yₜ*, *Y*_*t* +1_] relative to the size of category *i* at time *t*
*R*_*tin*_	The intensity of annual transition from category *i* to category *n* during the interval [*Yₜ*, *Y*_*t* +1_] relative to the size of category *i* at time *t*
*W*_*tn*_	The uniform intensity of annual transition from all non-*n* categories to category *n* during the interval [*γₜ* − *γₜ* + 1] relative to the size of all non-*n* categories at time *t*

1 $${S}_{t}=\frac{\text{Area of change during the interval }[{Y}_{t}, { Y}_{t+1}]}{(\text{Duration of the interval }[{Y}_{t}, { Y}_{t+1}] ) * (\text{Area of study region })}100{\%}$$

2 $$U =\frac{\text{Area of change during all interval}s}{\text{Duration of all intervals }* (\text{Area of study region }) }100{\%}$$

3 $${G}_{tj}=\frac{\text{Area of annual gain of category }j\text{ during the interval }[{Y}_{t}, { Y}_{t+1}]}{\text{Area of category }j\text{ at }{ Y}_{t+1}}100{\%}$$

4 $${L}_{ti}=\frac{\text{Area of annual loss of category }i\text{ during the interval }[{Y}_{t}, { Y}_{t+1}]}{\text{Area of category }i\text{ at }{Y}_{t}}100{\%}$$

5 $${R}_{tin} =\frac{\text{Area of annual transition from }i\text{ to n during the interval }[{Y}_{t}, { Y}_{t+1}]}{\text{Area of }i\text{ at }{Y}_{t}}100{\%}$$

6 $${W}_{tn}=\frac{\text{Area of annual gain of category n during the interval }[{Y}_{t}, { Y}_{t+1}]}{\text{Area of not category }n\text{ at }{Y}_{t}}100{\%}$$

At the interval level, the analysis evaluates the total change in every time interval, as well as both the observed change and the uniform rate of change. Equation (1) was used to calculate each interval’s annual rate of total change by dividing the transition size by the time interval to obtain a spatial extent percentage. Equation (2) was used to calculate the uniform rate, representing the change that would occur if annual changes were evenly distributed over the study period. Each interval’s annual change intensity is then compared to the uniform annual change rate. At the category level of Intensity Analysis, Eq. (3) was utilised to calculate the intensity of annual total gains for each land cover class, representing the percentage of additional surface area covered by that class. Conversely, Eq. (4) was used to calculate the intensity of annual gross loss, which indicates the percentage of surface area no longer occupied by the land cover class relative to its previously covered total area. The acquired annual gross gains and losses were then compared with the uniform intensity of change. Furthermore, the study evaluated variations in the intensity of transitions between land cover classes during each time interval. This stage involves evaluating the transition’s intensity toward a particular land cover class (gain) using Eq. (5) and assessing the transition’s intensity from a specific land cover class (loss) using Eq. (6). Transitions were analysed to determine whether they exhibited a preference for gaining from a certain land cover class or tended to avoid gaining from it.

### Cellular Automata model

The CA model was utilised to predict future land cover changes in the study area using the Modules for Land-Use Change Simulation (MOLUSCE) plugin in QGIS version 2.18.24. The MOLUSCE plugin was created to assess, model and simulate future LULC change (Aneesha Satya et al., 2020; Kamaraj & Rangarajan, 2022; Muhammad et al., 2022). The MOLUSCE plugin is also suitable for analysis of past LULC changes (Al-Rubkhi et al., 2017). It uses popular algorithms such as CA, Logistic Regression (LR) and ANN (Değermenci, 2023; Guidigan et al., 2019). The ANN algorithm and MLP-ANN learning process were employed for this study to model future land cover changes using six prediction phases namely, inputs, evaluation correlation, area change, transition potential modelling, MLP-ANN and validation.

**Fig. 3:**
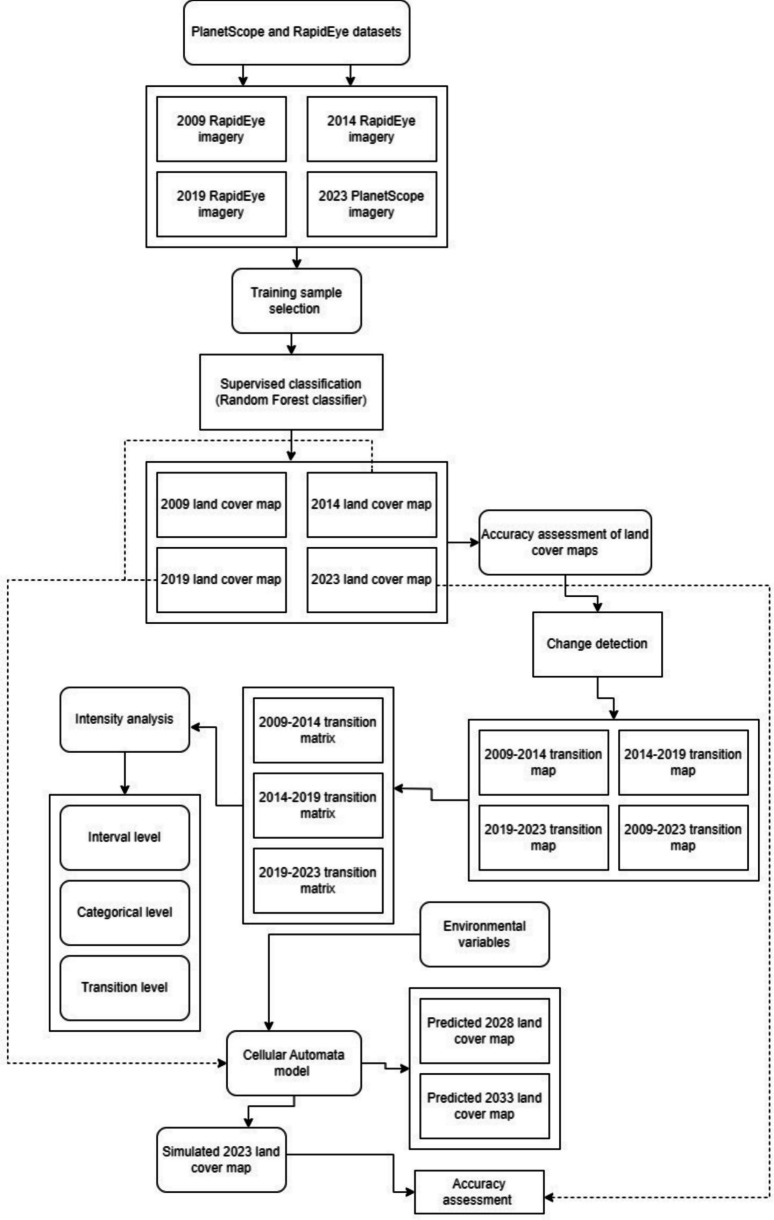
Methodology flowchart for land cover Intensity Analysis and the simulation of future bush encroachment

During the input phase, the classified maps for land cover change and environmental variables were inserted into the MOLUSCE plugin. All datasets were prepared and set to the same spatial resolution of 10 m and WGS 84 geographical coordinate system. For the first stage of the model, the 2014 classification map was used as the initial classified raster image, and 2019 was utilised as the final raster image. The initial and final raster images were then added to the model together with the environmental variables (DEM, slope, aspect, distance from road and Topographic Wetness Index) to prepare for the other stages of prediction of future bush encroachment trends. During the evaluation correlation phase, the model evaluates the correlation between two raster images or environmental variables using Pearson’s correlation, Cramer’s V coefficient or joint information uncertainty (Hakim et al., 2019). This study utilised Pearson’s correlation to test the correlation between driving factors. Subsequently, the model computed the area change in hectares from one land cover class to another between the initial (2014) and final year (2019). For the transition potential modelling stage, the model used the MLP-ANN learning process to simulate land cover transition potential (Hyandye & Martz, 2017; Kamaraj & Rangarajan, 2022). The method uses land cover change information and geographic factors as input to predict future trends of the investigated categories (Rahman et al., 2017). The Cellular Automata simulation phase was then utilised for the prediction and creation of future land cover maps using the MLP-ANN learning process. The study simulated the 2023 map using the 2014 and 2019 classification maps along with the selected environmental variables shown in Fig. 2. The accuracy of the model was evaluated through the model’s validation process, which involved comparing the overall per cent of correctness and kappa value of the reference (classified 2023) and simulation (simulated 2023) maps. After completing all six stages of the simulation process using the CA model, raster maps for 2028 and 2033 depicting simulated bush encroachment trends were generated (Fig. 3).

## Results

### Classification accuracy

**Fig. 4:**
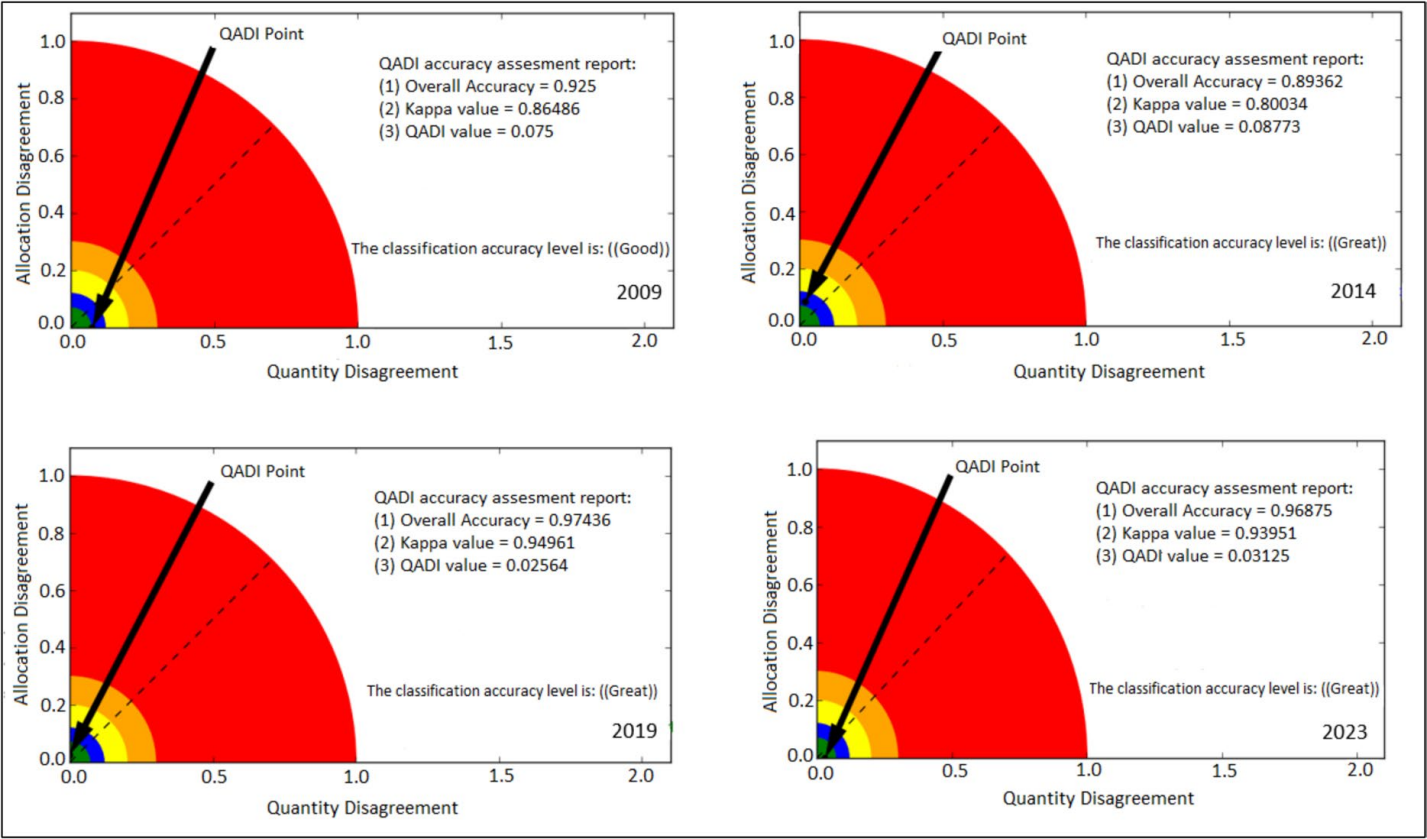
QADI graph showing the classification accuracy as a black dot

The study achieved an overall accuracy exceeding 85% for all image classifications. Specifically, high overall accuracies of 92.5, 89.4, 97.4 and 96.9% were obtained in 2009, 2014, 2019 and 2023, respectively. The study also yielded good kappa values, with the highest values recorded in 2019 (95.0%) and 2023 (94.0%), while relatively lower values were observed in 2009 (86.5%) and 2014 (80.0%). Additionally, the QADI index demonstrated good classification accuracy, as all image classifications yielded QADI values close to zero, suggesting minimal disagreement between the training data and classification results. The QADI values recorded were 0.075 in 2009, 0.088 in 2014, 0.026 in 2019 and 0.031 in 2022, further confirming the reliability of the classification outcomes (Fig. 4).

Appendix 2 (tables) shows the confusion matrices for different periods. In each confusion matrix, the last two columns indicate the total number of training set pixels that were correctly and incorrectly classified into their respective LULC classes. Omission and commission errors for each land cover are represented by the highlighted numbers under the class. Specifically, omission errors denote the number of pixels of a particular class that were misclassified into another class. For instance, in Appendix 2 (Table 5), the results indicate that 2 pixels from the woody vegetation class were incorrectly classified as grasses, while 0 pixels were incorrectly classified as bare areas. This results in a total omission error of 2 pixels for woody vegetation. For grasses, 0 pixels were misclassified as woody vegetation or bare areas, leading to a total omission error of 0 pixels. Additionally, the results show that 1 pixel from the bare area class was misclassified as woody vegetation, while 2 pixels were misclassified as grasses.

Conversely, commission errors represent the number of pixels incorrectly classified as belonging to a particular class when they are not. As shown in Appendix 2 (Table 5), for example, 0 pixels of woody vegetation were misclassified as grasses, while 1 pixel was misclassified as a bare area, resulting in a total commission error of 1 for woody vegetation. Similarly, 0 pixels of grasses were misclassified as woody vegetation, whereas 2 pixels were misclassified as bare areas, leading to a total commission error of 2 for grasses. For bare areas, 0 pixels were misclassified as either woody vegetation or grasses, yielding a total commission error of 0 for grasses. Overall, these accuracy results demonstrate that the classification outputs are reliable and suitable for deriving critical insights into bush encroachment trends.

### Bush encroachment between 2009 and 2023

**Fig. 5:**
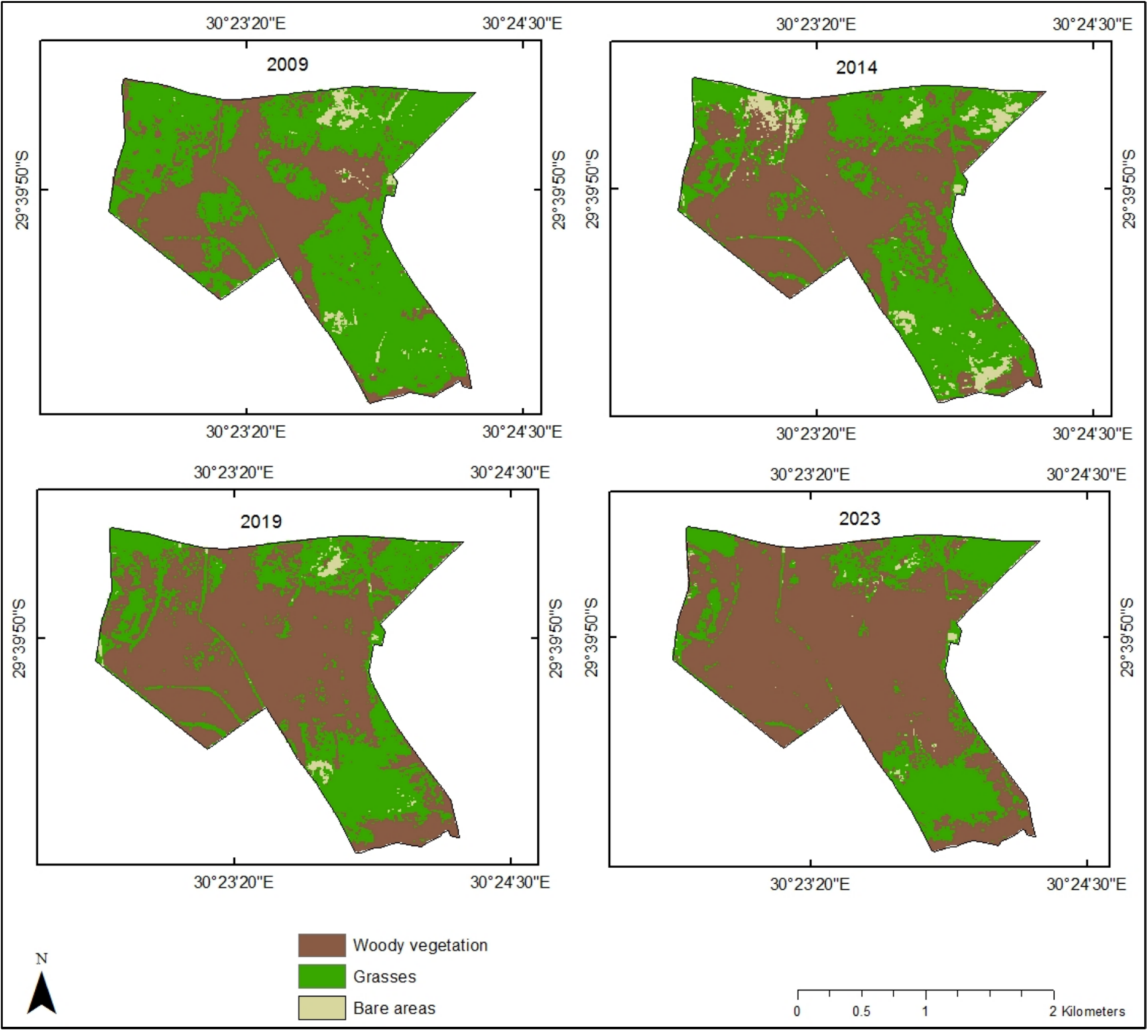
Classification map showing the progression of woody vegetation in Bisley Nature Reserve during the study period

The results from the classification maps and change matrix show increasing bush encroachment over the study period, as illustrated in Fig. 5 and Table 2. Over the 14-year period, bush encroachment expanded by approximately 128.54 ha (36.33%), whilst grassland areas decreased by around 123.36 ha (34.87%). The proportion of land covered by woody vegetation, grasses and bare areas varied across different periods. Initially, grasses were the dominant land cover class, occupying approximately 213.15 ha (60.25%) of the study area. However, in subsequent years, woody vegetation became the dominant class, covering 51.18% in 2014, 64.52% in 2019 and 74.02% in 2023.

**Table 2:** Area (ha) and percentage cover of woody vegetation, grasses and bare areas in Bisley Nature Reserve

Classes	2009		2014		2019		2023	
	Area	%cover	Area	%cover	Area	%cover	Area	%cover
Woody vegetation	133.32	37.69	181.05	51.18	224.55	64.52	261.86	74.02
Grasses	213.15	60.25	153.79	43.47	124.93	35.32	89.79	25.38
Bare areas	7.28	2.06	18.96	5.35	4.28	0.16	2.00	0.60

### Change statistics

**Fig. 6:**
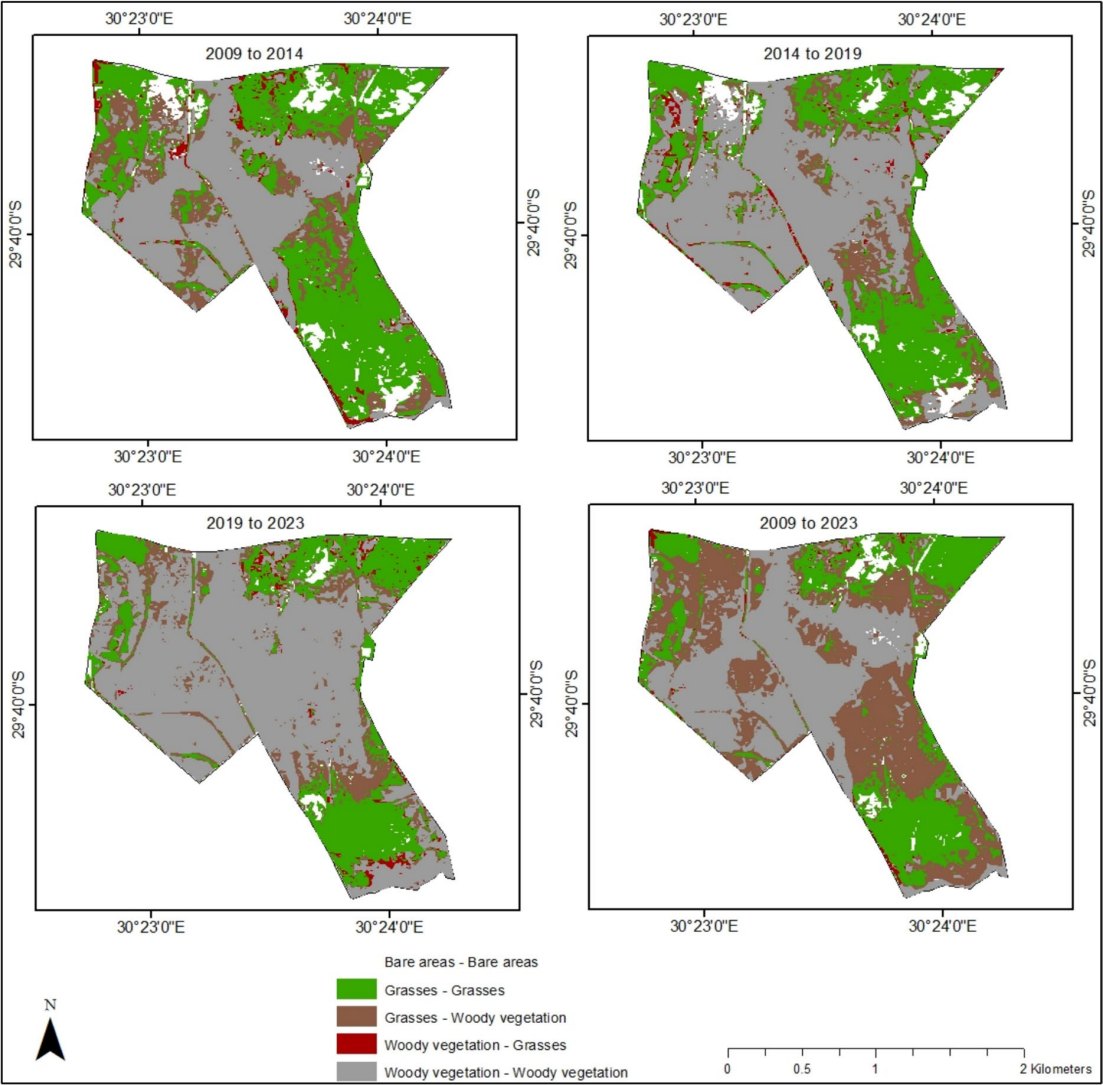
Change detection map of Bisley Nature Reserve showing the transition of woody vegetation and grasses for different time periods

The land cover transitions were computed to determine gains and losses in different land cover classes over time (Fig. 6; Table 3). The findings of the study demonstrate that the nature reserve is progressively threatened by bush encroachment. However, the extent of encroachment showed a general decline throughout the study period. Notably, the transition of grasses to woody vegetation decreased by 19.43% between the 2009–2014 and 2014–2019 periods, followed by a minimal decline of 6.65% from 2014–2019 to the next period (2019–2023). Overall, 36.94% of the grassy areas transitioned to woody vegetation during the study period, whilst only 0.78% of the woody vegetation area was converted back to grasses. Additionally, a portion of the grassland was lost to bare areas, with a significant 4.54% reduction recorded during the 2009–2014 period.

**Table 3:** Transitions and area change in hectares (ha) between woody vegetation, grasses and bare areas in Bisley Nature Reserve

Classes	Area change (ha)			
	2009–2014	2014–2019	2019–2023	2009–2023
Grasses to grasses	137.98	104.85	97.19	80.52
Grasses to woody vegetation	58.63	47.24	44.10	130.69
Woody vegetation to grasses	10.18	8.74	6.96	2.78
Woody vegetation to woody vegetation	121.70	172.06	217.23	130.07
Grasses to bare areas	16.07	1.41	1.08	0.43
Bare areas to grasses	5.21	10.10	3.41	6.2

### Intensity Analysis

**Fig. 7:**
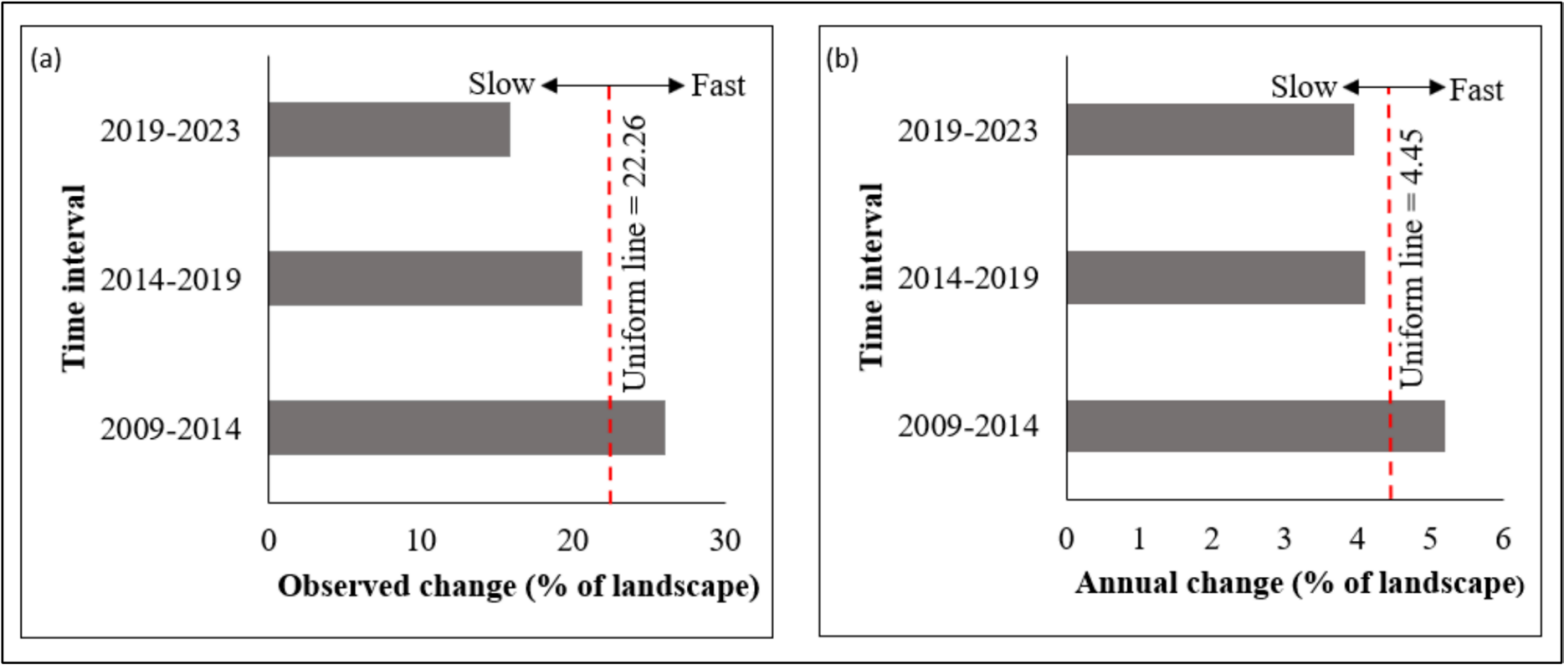
Interval-level intensity of Bisley land cover class changes for three periods. **a** The percentage of area that changed in each period and **b** the percentage of annual area changes in each period

Figure 7 presents the interval-level intensity findings, where Fig. 7a illustrates the observed change intensity across different time periods, and Fig. 7b depicts the annual change in area between these periods. The study findings indicate that the 2009–2014 period experienced a rapid annual change intensity (5.20% per year compared to a uniform intensity of 4.45%) and a high intensity/transition rate (25.98% per period against a uniform intensity of 22.26%). In contrast, during the subsequent intervals (2014–2019 and 2019–2023), both the annual change intensity and transition rates remained below the uniform line, suggesting slower annual rates of change and transitions during these periods.

**Fig. 8:**
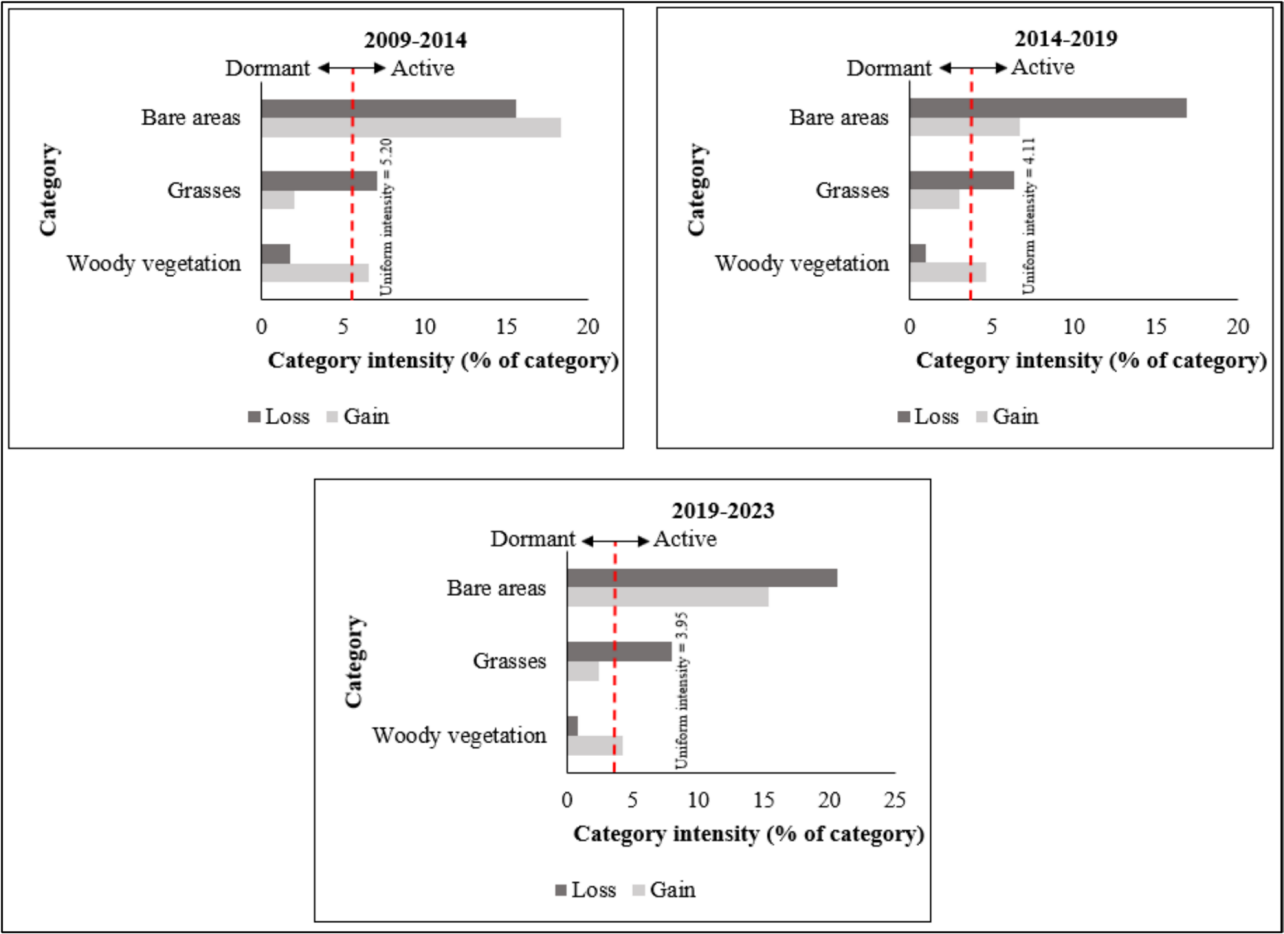
Category-level intensity of land cover changes for three different periods

The category-level analysis graphs (Fig. 8) illustrate the gross losses and gains amongst land cover classes, identifying active and dormant land cover classes for each period. A land cover class is considered active when its gains or losses exceed the uniform intensity line, whereas, in a dormant category, they fall below this threshold. Throughout all periods, bare areas and woody vegetation were active gainers, whilst grasses remained the dormant gainer. The bare area class experienced the highest gains across all time periods, although these gains fluctuated—decreasing from the first to the second period but increasing in the third. Meanwhile, woody vegetation gains showed a slight decline across successive periods, with the highest class gain occurring during the first period.

Bare areas and grasslands consistently experienced net losses throughout the three time intervals. Notably, bare areas were the only category that exhibited both gains and losses across all three periods. The highest losses in the bare area class occurred during the 2014–2019 and 2019–2023 periods. Meanwhile, grassland losses decreased from the first period to the next but increased in the final period. The category-level analysis findings further indicate that woody vegetation remained the dormant loser throughout all three periods.

Appendix 1 presents the transition-level Intensity Analysis results for the three land cover classes. The gains and losses of each category are explained based on uniform intensity lines, which indicate the transitions’ uniform intensity values. The lines on the left side represent losses in a particular land cover class, whilst those on the right side depict class gains. The transition Intensity Analysis results reveal that during the first two periods (2009–2014 and 2014–2019), woody vegetation predominantly gained from grasses. However, in the 2019–2023 period, woody vegetation gained less intensively from grasses, with gains slightly lower than the uniform intensity value. Additionally, the results indicate that during the 2009–2014 period, woody vegetation experienced minimal gains from and losses to bare areas. During the 2009–2014 period, losses in bare areas were more intensive from grasslands, whilst grassland gains were also intensive from bare areas but less prevalent from woody vegetation. Moreover, grassland losses to woody vegetation were minimal during this period. A similar pattern was observed in the subsequent periods, where transitions to grassland primarily occurred from bare areas. The results further indicate that during the 2014–2019 and 2019–2023 periods, both gains and losses for bare areas were intensive from grasslands, whereas gains for grassland were predominantly from bare areas.

### Prediction of future bush encroachment

**Fig. 9:**
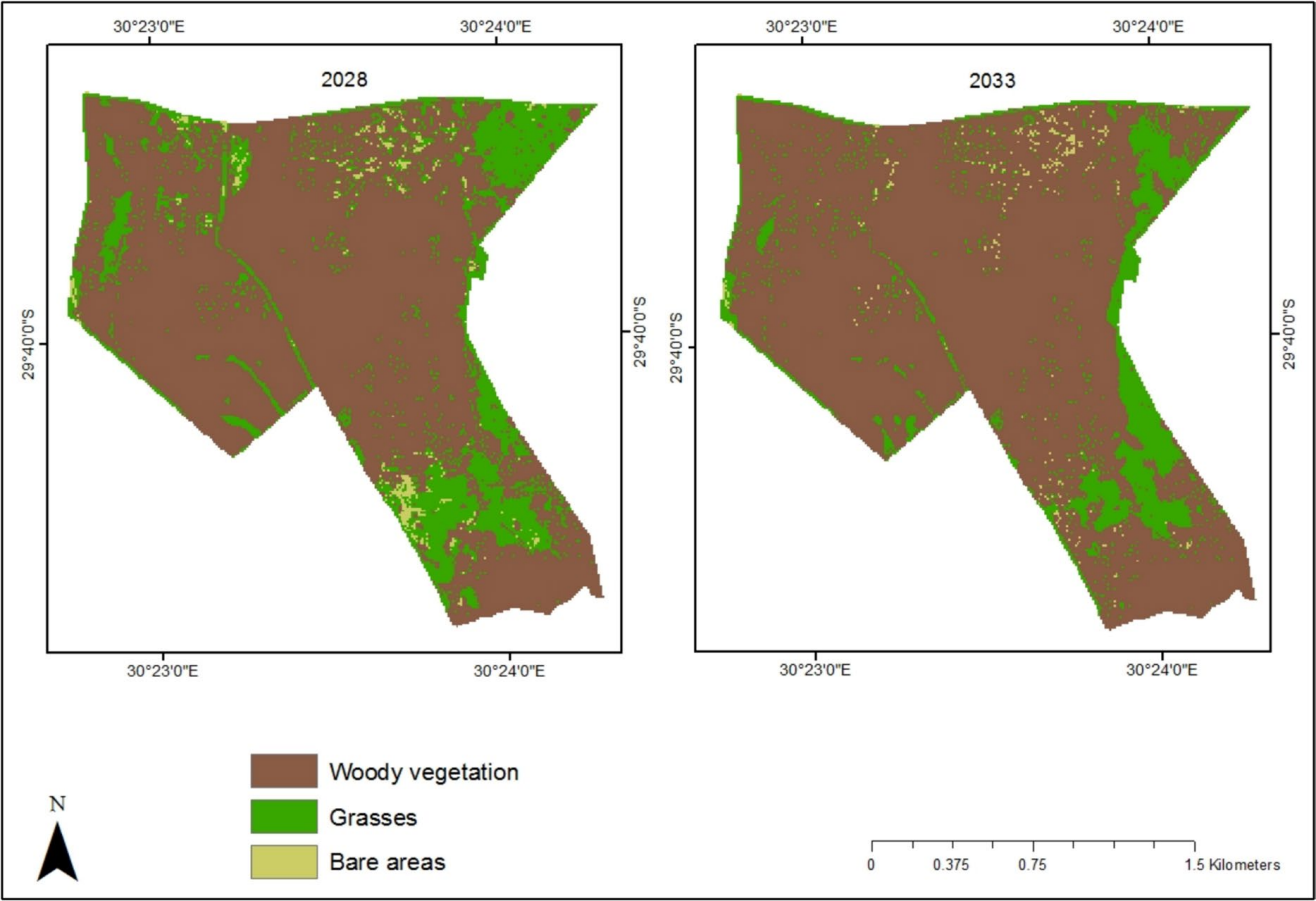
Simulation map showing the future progression of woody vegetation in Bisley Nature Reserve for 2028 and 2033

**Fig. 10:**
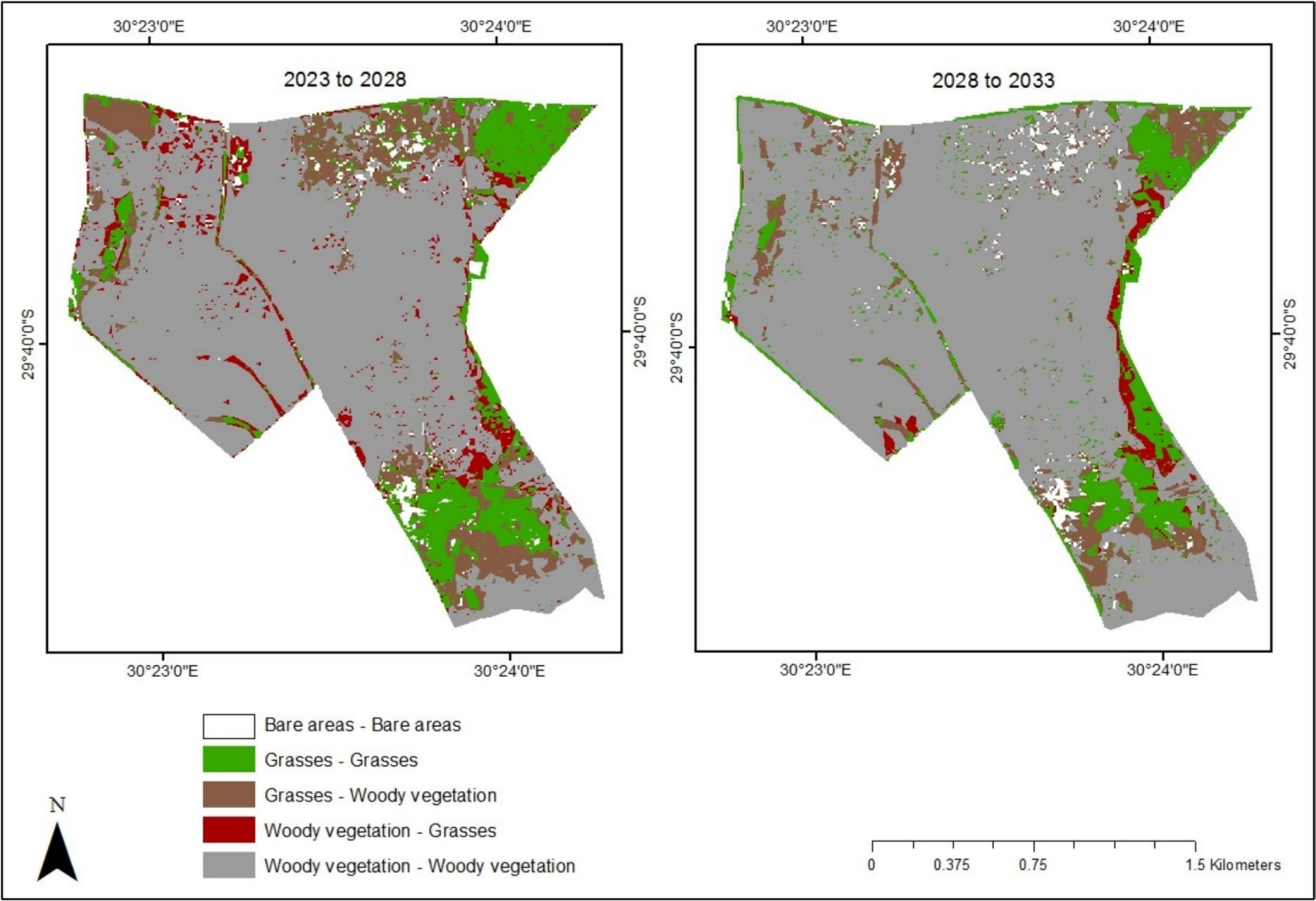
Predicted transition map of Bisley Nature Reserve showing the transition of woody vegetation and grasses for different time periods

The current study simulated the percentage cover for woody vegetation, grasses and bare areas in Bisley Nature Reserve for the years 2028 and 2033. The CA model predicted a minimal expansion of woody vegetation from the current year (2023) to 2028 and 2033. Specifically, woody vegetation is expected to increase from 261.86 ha (74.02%) in 2023 to 281.31 ha (79.52%) and 304.91 ha (86.19%) in 2028 and 2033, respectively. During the first five-year period (2023–2028), only 5.50% of land is projected to transition to woody vegetation. However, for the next five years (2028–2033), the percentage area covered by woody vegetation is anticipated to rise by 6.67%. The model also indicates that Bisley’s grass cover will decline from 89.79 ha (25.38%) in 2023 to 66.89 ha (18.91%) in 2028 and further decrease to 46.10 ha (13.03%) by 2033. Additionally, the results show that bare areas will expand from 2.00 ha (0.60%) in 2023 to 5.55 ha (1.57%) in 2028 before decreasing to 2.75 ha (0.78%) by 2033 (Figs. 9 and 10).

### Accuracy of bush encroachment predictions

**Fig. 11:**
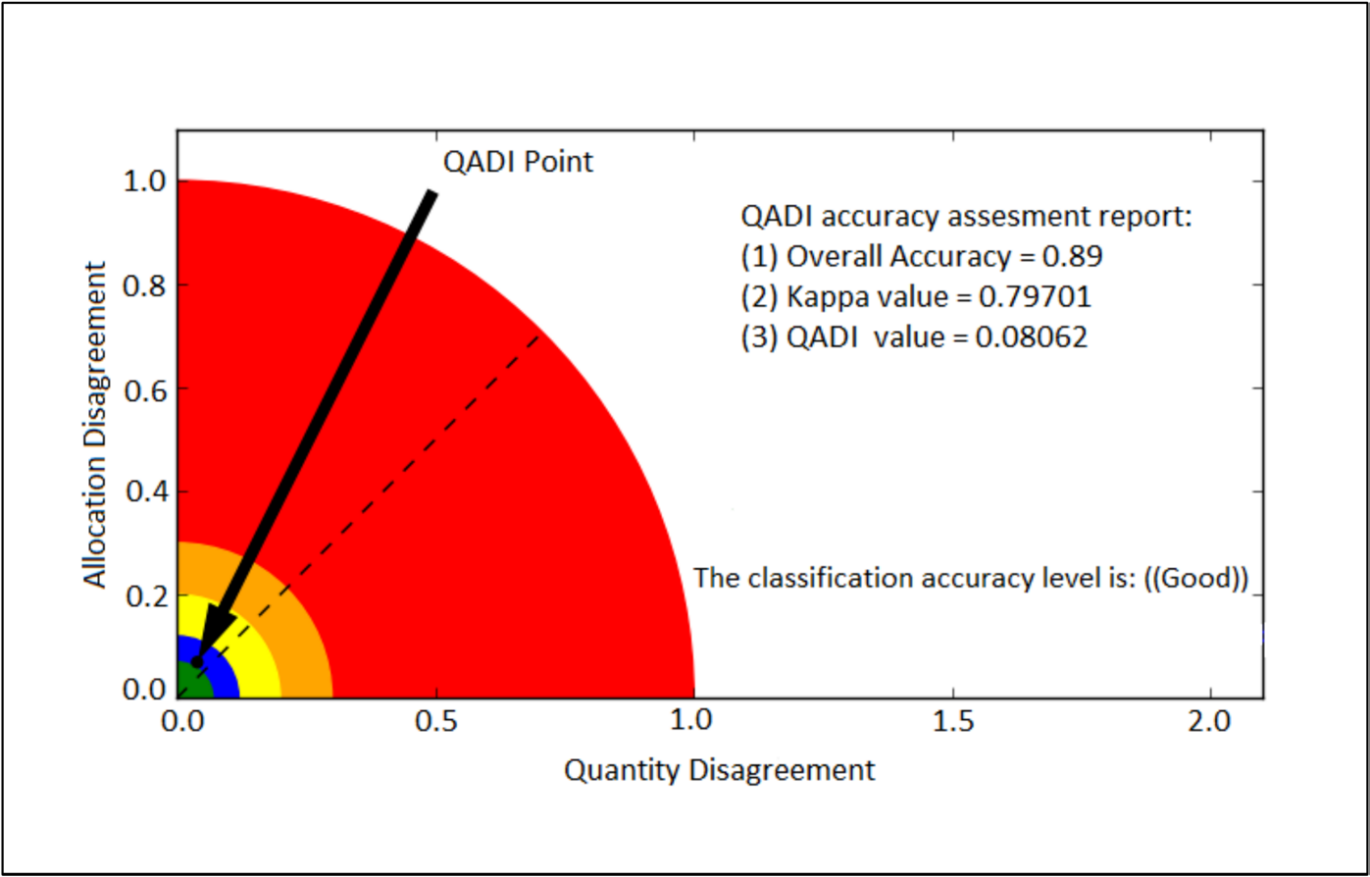
QADI graph showing model simulation accuracy for 2023 as a black dot

The model accuracy was assessed by comparing the reference (classified 2023) and simulation (simulated 2023) maps. The accuracy results demonstrated that the model effectively predicted the future encroachment trends in Bisley Nature Reserve. The model simulated land cover transitions with an overall accuracy of 89%. The study also yielded good kappa values of 78%. Additionally, the QADI index indicated good accuracy, attaining a QADI value of 0.08 (Fig. 11). The black dot is positioned above the dotted diagonal line and near the allocation disagreement axis, indicating that the primary factor contributing to the observed minimal disagreement is the allocation error. Overall, the achieved accuracy results demonstrate that the model prediction is reliable and that the reference and prediction maps are comparable.

## Discussion

The analysis of landscape changes, particularly the encroachment of bushes into grasslands within protected areas, is crucial for effective management to ensure the preservation of natural ecosystems, biodiversity and recreational activities. Hence, this study sought to provide a comprehensive analysis of bush encroachment in Bisley Nature Reserve by mapping its progression in the landscape, assessing the rate of change and intensity of transitions and predicting future bush encroachment patterns.

### Bush encroachment in the study area

The analysis of bush encroachment trends over the past 14 years revealed that Bisley Nature Reserve experienced woody vegetation expansion at the expense of grassland. Throughout the study period, woody vegetation increased by 36.33%, leading to an approximately 34.87% reduction in grassland. The study suggests that this expansion of woody vegetation is associated with disturbances such as climate change and rainfall variability, the presence of alien invasive species and the application of injudicious land management approaches, including infrequent fires and inconsistent mechanical control methods (Kellner et al., 2022; Liao et al., 2018). The study area is also characterised by unstable climatic conditions and heavy rains, which may promote the overgrowth of woody vegetation (Ndlovu & Demlie, 2020). Moreover, the nature reserve is invaded by invasive woody plants such as *Acacia* spp., *Solanum mauritianum* and *Lantana camara* (Kraai, 2021). These species are known to proliferate rapidly in newly established landscapes (Gazoulis et al., 2022).

### Bush encroachment Intensity Analysis

The results from the interval-level analysis showed an intensive change in Bisley’s land cover during the first period (2009–2014) compared to the successive periods (2014–2019 and 2019–2023). However, change during the 2019–2023 period was slower than during 2014–2019. This implies that during the three time periods, the study area had been subjected to transformations at different rates, with a rapid transformation observed during the first five years of the study period. The rapid transformations in Bisley land cover and change in the intensity of transitions are hypothesised to be influenced by improvements in the management of the reserve and increasing concerns about biodiversity conservation. According to Kourosh Niya et al. (2019), for instance, the intensity of land cover changes is associated with developments and advancements of management schemes in a particular landscape. Other studies on the Intensity Analysis of land cover have also reported rapid land cover change in the first and second intervals compared to subsequent intervals, indicating that the rate of changes in the area was progressing steadily in recent periods (Bogale et al., 2024; Feng et al., 2020; Osman et al., 2023; Sang et al., 2019). The slowing intensity of land cover transitions in recent periods could also be associated with infilling, where the availability of open grassland for woody vegetation encroachment decreases over time.

The categorical-level analysis revealed that woody vegetation and bare areas were the primary gainers during the study period. This trend is hypothesised to be influenced by climate change and rainfall variability, which promote the growth and dominance of woody vegetation whilst also contributing to grassland degradation through erosion (Kraai et al., 2022). Notably, bare areas were the only category that experienced both active gains and active losses across all three periods. These findings indicate that changes in bare areas occurred at intensities higher than the average intensity of gains and losses across all land cover classes (Osman et al., 2023; Ouedraogo et al., 2023). The intensities of active gains in the bare area class are attributed to landscape disturbances, particularly burrowing by smaller mammals such as porcupines and the effects of heavy rains and floods (Centeri, 2022; Kraai et al., 2023). Conversely, active losses in this class are linked to management and restoration schemes (D’Odorico et al., 2012; Maphanga et al., 2022). The findings also indicate that across all periods, grasses were the dormant gainer and active loser due to suppression by the overgrowth of woody vegetation and the effects of heavy rainfall and soil erosion. Interestingly, the categorical analysis revealed that woody vegetation gains slightly declined in each successive period, with class gains of 6.54% during 2009–2014, decreasing to 4.66% in 2014–2019 and 4.23% in 2019–2023. These findings suggest that certain management schemes, such as mechanical clearing of alien invasive woody plant species and controlled winter fires, have had a minimal yet observable impact in curbing woody vegetation overgrowth.

The key findings from the transition-level analysis indicate that woody vegetation gains from grasslands were higher during the 2014–2019 period compared to the 2009–2014 period. The major expansion of woody vegetation at the expense of grasslands during the second period is attributed to inconsistent bush encroachment control measures at Bisley and rainfall variability, which may have triggered rapid overgrowth of woody vegetation in certain years (Kraai et al., 2022; Makin et al., 2012). The findings also show that during the 2019–2023 period, woody vegetation exhibited less intensive gains and losses to grasslands. These results suggest that the increase in woody vegetation contributed to a decline in grasslands at Bisley grasses and that woody vegetation gains from grasslands fluctuated across different periods. The observed low-intensity gains and losses of woody vegetation on grasslands during the recent period (2019–2023) are hypothesised to be associated with advancements in management schemes, particularly controlled winter fires and bush clearing, as well as a growing interest in conserving biodiversity and the recreational value of Bisley Nature Reserve. Additionally, woody vegetation exhibited minimal gains from and losses to bare areas across all periods. These findings suggest that woody vegetation had a limited role in the transformation of land into bare areas and that bare areas are less conducive to the establishment, invasion and dominance of woody vegetation in Bisley. The findings also showed that, across all periods, gains in grasses primarily originated from bare areas. This trend is attributed to the relatively straightforward and rapid process of grassland restoration from bare areas to the more complex and resource-intensive conversion of woody landscapes to grassland areas, which may take many years (Kellner et al., 2021; Mndela et al., 2022). An interesting key finding from the Intensity Analysis at the transition level is that woody vegetation gains were more intensive on grasslands, whereas grasslands were consistently lost to bare areas across all periods.

### Bush encroachment future outlooks

The results of the study demonstrate that by 2028, bush encroachment will increase by 5.50 and 6.67% by 2033. The predicted increase in woody vegetation is hypothesised to be linked to climate change and rainfall variability. For instance, in recent years, including 2023 and 2024, South Africa has experienced climate variability and change, characterised by heavy rains and storms (Mashao et al., 2023; Sivakumar & Fazel-Rastgar, 2021). There is a possibility of continued rainfall variability that will induce the growth and expansion of woody vegetation in Bisley (Brandt et al., 2017). Additionally, the observed trend of woody vegetation expansion in the area may be influenced by the adaptation and overgrowth of invasive plant species, particularly *acacia* species. According to Kaplan et al. (2012), *acacia* species are amongst the most pernicious and prolific invasive plant species in many regions, including Southern Africa. These species affect the quality and quantity of grasslands and soil resources and are frequently reported as drivers of ecosystem degradation in the grasslands of South Africa (Jansen & Kumschick, 2022; Yapi et al., 2018). The model also indicates that the trend of grassland loss to woody vegetation in Bisley will continue over the next decade, with approximately 40.68 ha of grasses lost from 2023 to 2028 and an additional 28.97 ha lost in the following period (Table 4). The research findings highlight the need to evaluate grass management schemes to ensure effective reserve management and achieve conservation goals.

**Table 4:** Transitions and area change in hectares (ha) between woody vegetation, grasses and bare areas in Bisley Nature Reserve

Classes	Area change (ha)	
	2023–2028	2028–2033
Grasses to grasses	44.84	37.07
Grasses to woody vegetation	40.68	28.97
Woody vegetation to grasses	20.87	8.41
Woody vegetation to woody vegetation	239.39	272.55
Grasses to bare areas	3.99	0.86
Bare areas to grasses	0.64	0.61

The prediction of future bush encroachment trends could guide landscape management in Bisley Nature Reserve and present viable strategies for effective biodiversity conservation. Prediction results will enhance understanding of the implications of bush encroachment for various species within the reserve (Gambo et al., 2018; Teferi, 2021). The approach will provide insights into habitat loss and be crucial for understanding ecological succession and the resilience of the Bisley ecosystem to shifts in vegetation cover and structure (Liao et al., 2018; Sahara et al., 2015). Consequently, it will also provide a deeper understanding of the potential impacts of bush encroachment on the balance between grazer and browser species within the reserve, as well as its implications for ecosystem services, particularly carbon sequestration, erosion control and recreational opportunities for Bisley and the Pietermaritzburg community (Kusiima et al., 2022; O’Connor et al., 2014). Furthermore, understanding future bush encroachment prospects could help assess its impact on local hydrological processes and water availability, as well as the influence of invasive species (Rolo & Moreno, 2019).

### Ecological and management implications of the study

Based on the results of the study, various ecological and management implications were identified to highlight the importance of spatiotemporal analysis of bush encroachment using approaches such as change detection, Intensity Analysis and the prediction of future encroachment. Such spatially explicit and detailed analyses provide essential insights into the dynamics and major factors influencing the invasion and overgrowth of woody vegetation in landscapes such as nature reserves (Mndela, 2020; Rolo & Moreno, 2019). The findings of the study are particularly essential for promoting the sustainable conservation of biodiversity and recreational activities. They can profoundly influence the evaluation and improvement of management schemes, contributing to the proper management and restoration of nature reserves and other protected landscapes. The analysis of landscape changes is also pivotal for maintaining habitat heterogeneity and healthy ecosystems (Phan et al., 2020). Additionally, it contributes to the prevention of natural catastrophes related to bush encroachment (Skowno et al., 2017; Thomas et al., 2018). According to Sobhani et al. (2021), research on landscape design and land use significantly contributes to sustainable land use and the proper management of protected areas, reducing erosion risks and other forms of land degradation.

### Limitations of the study

Generally, the full maturation of woody vegetation can take three to ten years. As a result, a longer study period is recommended for assessing bush encroachment trends and progression. An extended period is paramount for explicitly identifying and quantifying spatial changes in the landscape, enabling improved landscape management and restoration. This study examined bush encroachment dynamics in a nature reserve over the past 14 years. The selection of a 14-year study period was particularly influenced by data accessibility and availability. Bisley Nature Reserve has a small spatial extent of approximately 350 ha; consequently, high spatial resolution remotely sensed data were required for monitoring bush encroachment in the area. However, high spatial resolution datasets such as PlanetScope and RapidEye were launched in 2014 and 2008, respectively. As a result, the study was limited to a 14-year study period. Moreover, the RapidEye constellation was decommissioned in December 2019, posing a significant challenge in acquiring recent data for change detection analysis. To address this, PlanetScope was used to supplement and provide recent image data. The study utilized environmental but not climatic variables for predicting future bush encroachment. This limitation was due to the small spatial extent of the study site (350 ha) and challenges in acquiring high-resolution climate data specific to such a localized scale. Most available climate datasets are designed for regional or larger spatial scales and may not accurately capture microclimatic variations relevant to the study site. Consequently, using such climate variables could undermine the model’s accuracy. Additionally, the prediction of future bush encroachment cannot be directly linked to increasing climate variability, as the model did not incorporate climate data to assess its impact on bush encroachment in the study area. Another limitation of the study was the lack of detailed records on management practices in Bisley Nature Reserve; therefore, management practices were not included as variables in the model prediction. Furthermore, the study adopted the commonly used majority-based LULC classification method, which reduces noise, prevents the influence of outliers and produces reliable land cover maps. However, a limitation of this method is that it assigns pixels to a single class, and potentially overlooks subpixel heterogeneity.

## Conclusion

Understanding historical and future trends, as well as the progression of bush encroachment, is critically important for the management and restoration of grasses in protected areas. This study utilized an Intensity Analysis framework to develop insights into the size and intensity of bush encroachment transitions in Bisley Nature Reserve. Moreover, it predicted future bush encroachment outlooks using the Cellular Automata model. The results revealed that Bisley Nature Reserve is undergoing a transformation in which its grasses are continuously threatened by bush encroachment. Based on these findings, it is concluded that:

1. The use of robust quantitative methods, such as Intensity Analysis, facilitates an understanding of the dynamics of bush encroachment and provides explicit information on landscape changes.
2. Bush encroachment is increasing in Bisley Nature Reserve, with over 85% of grasses expected to be encroached by 2033.

The use of the Intensity Analysis framework provides additional insights into bush encroachment that were not feasible to acquire using a general transition matrix and change detection techniques. Specifically, this method is valuable for offering critical information on the intensity of transitions and annual rates of change, which could aid in improving management and conservation initiatives. Moreover, predicting future bush encroachment using the Cellular Automata model further enhances the explicit understanding of encroachment trends and the vulnerability of grasslands. This study is invaluable to conservation initiatives and provides a foundation for the proper evaluation and implementation of effective management schemes in protected areas and other grassy landscapes.

## Figures and Tables

**Fig. 12:**
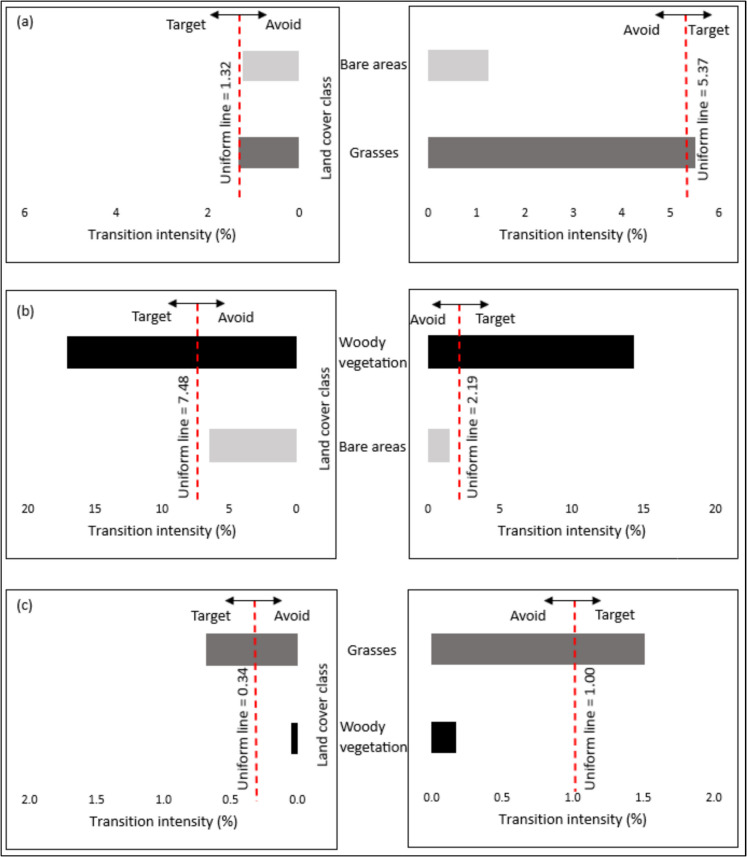
Period 2009–2014 transition-level Intensity Analysis. **a** Transition intensity for woody vegetation, **b** grasses and **c** bare areas (gains on the right and losses on the left)

**Fig. 13:**
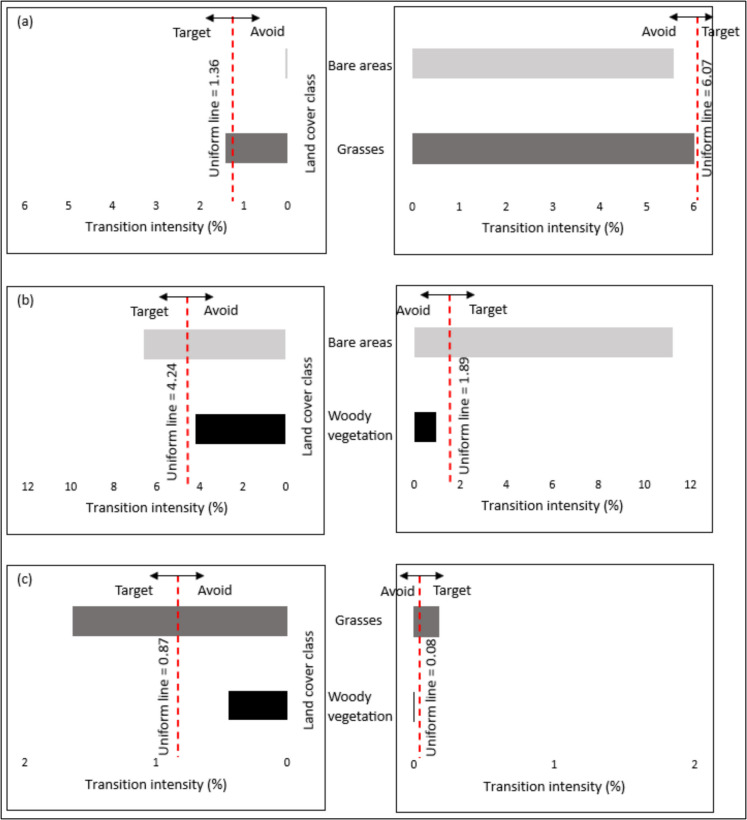
Period 2014–2019 transition-level Intensity Analysis. **a** Transition intensity for woody vegetation, **b** grasses and **c** bare areas (gains on the right and losses on the left)

**Fig. 14:**
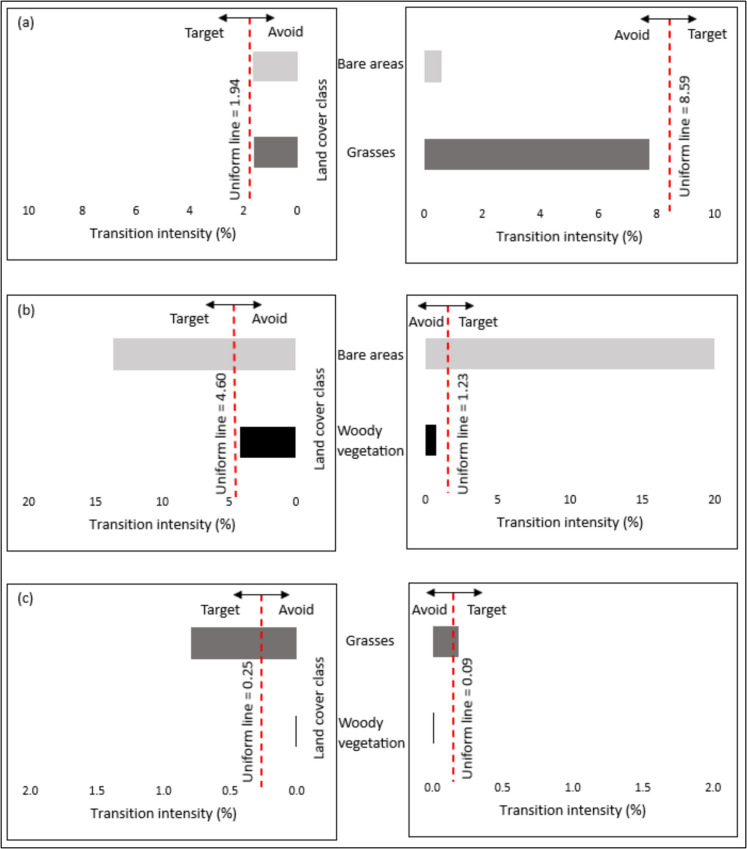
Period 2019–2023 transition-level Intensity Analysis. **a** Transition intensity for woody vegetation, **b** grasses and **c** bare areas (gains on the right and losses on the left)

## Data Availability

No datasets were generated or analysed during the current study.

## Appendix 1

Figures 12, 13, and 14

## Appendix 2

Tables 5, 6, 7, and 8

**Table 5:** Confusion matrix for 2009 classification

Classes	Woody vegetation	Grasses classified	Bare areas classified	Total correctly	Total incorrectly
Woody vegetation	20	0	0	20	0
Grasslands	0	16	0	16	0
Bare areas	1	2	1	1	3

**Table 6:** Confusion matrix for 2014 classification

Classes	Woody vegetation	Grasses classified	Bare areas classified	Total correctly	Total incorrectly
Woody vegetation	16	2	0	16	2
Grasses	2	24	1	24	3
Bare areas	0	0	2	2	0

**Table 7:** Confusion matrix for 2019 classification

Classes	Woody vegetation	Grasses classified	Bare areas classified	Total correctly	Total incorrectly
Woody vegetation	22	0	0	22	0
Grasses	1	15	0	15	1
Bare areas	0	0	1	1	0

**Table 8:** Confusion matrix for 2023 classification

Classes	Woody vegetation	Grasses classified	Bare areas classified	Total correctly	Total incorrectly
Woody vegetation	19	0	0	19	0
Grasses	0	11	1	11	1
Bare areas	0	0	1	1	0

## Appendix 3

Table 9

**Table 9:** Field data summary

Encroachment category	Transects (70 m)	Sample plots (25 m^2^; 10 m apart)	GPS location (per plot)
Heavy encroached	10	50	1 for each LULC class
Medium encroached	10	50	1 for each LULC class
No encroachment	10	50	1 for each LULC class
Total	30	150	150 GPS locations for each LULC class

## Appendix 4

GEE Script link: https://code.earthengine.google.com/82c72cc36bd6eb027928b1c81f8fbfaf.
